# Mutational landscape and genetic signatures of cell‐free DNA in tumour‐induced osteomalacia

**DOI:** 10.1111/jcmm.14991

**Published:** 2020-04-11

**Authors:** Nan Wu, Zhen Zhang, Xi Zhou, Hengqiang Zhao, Yue Ming, Xue Wu, Xian Zhang, Xin‐Zhuang Yang, Meng Zhou, Hua Bao, Weisheng Chen, Yong Wu, Sen Liu, Huizi Wang, Yuchen Niu, Yalun Li, Yu Zheng, Yang Shao, Na Gao, Ying Yang, Ying Liu, Wenli Li, Jia Liu, Na Zhang, Xu Yang, Yuan Xu, Mei Li, Yingli Sun, Jianzhong Su, Jianguo Zhang, Weibo Xia, Guixing Qiu, Yong Liu, Jiaqi Liu, Zhihong Wu

**Affiliations:** ^1^ Department of Orthopedic Surgery Peking Union Medical College Hospital Peking Union Medical College and Chinese Academy of Medical Sciences Beijing China; ^2^ Beijing Key Laboratory for Genetic Research of Skeletal Deformity Beijing China; ^3^ Key laboratory of big data for spinal deformities Chinese Academy of Medical Sciences Beijing China; ^4^ School of Biomedical Engineering School of Ophthalmology and Optometry and Eye Hospital Wenzhou Medical University Wenzhou China; ^5^ PET‐CT Center National Cancer Center/National Clinical Research Center for Cancer/Cancer Hospital Chinese Academy of Medical Sciences and Peking Union Medical College Beijing China; ^6^ Translational Medicine Research Institute Geneseeq Technology Inc. Toronto ON Canada; ^7^ Nanjing Geneseeq Technology Inc. Nanjing China; ^8^ Department of Central Laboratory Peking Union Medical College Hospital Peking Union Medical College and Chinese Academy of Medical Sciences Beijing China; ^9^ Graduate School of Peking Union Medical College Beijing China; ^10^ Department of Breast Surgery The Affiliated Yantai Yuhuangding Hospital of Qingdao University Yantai China; ^11^ Beijing Ekitech Co. Ltd. Beijing China; ^12^ Department of Endocrinology Key Laboratory of Endocrinology Ministry of Health Peking Union Medical College Hospital Peking Union Medical College and Chinese Academy of Medical Sciences Beijing China; ^13^ Key Laboratory of Genomic and Precision Medicine China Gastrointestinal Cancer Research Center Beijing Institute of Genomics Chinese Academy of Sciences Beijing China; ^14^ University of Chinese Academy of Sciences Beijing China; ^15^ Department of Breast Surgical Oncology National Cancer Center/National Clinical Research Center for Cancer/Cancer Hospital Chinese Academy of Medical Sciences and Peking Union Medical College Beijing China

**Keywords:** cell‐free DNA, fibroblast growth factor receptor‐1, mediator complex subunit 12, next‐generation sequencing, tumour‐induced osteomalacia

## Abstract

Tumour‐induced osteomalacia (TIO) is a very rare paraneoplastic syndrome with bone pain, fractures and muscle weakness, which is mostly caused by phosphaturic mesenchymal tumours (PMTs). Cell‐free DNA (cfDNA) has been regarded as a non‐invasive liquid biopsy for many malignant tumours. However, it has not been studied in benign tumours, which prompted us to adopt the targeted next‐generation sequencing approach to compare cfDNAs of 4 TIO patients, four patients with bone metastasis (BM) and 10 healthy controls. The mutational landscapes of cfDNA in TIO and BM groups were similar in the spectrum of allele frequencies and mutation types. Markedly, deleterious missense mutations in *FGFR1* and loss‐of‐function mutations in *MED12* were found in 3/4 TIO patients but none of BM patients. The gene ontology analysis strongly supported that these mutated genes found in TIOs would play a potential role in PMTs' process. The genetic signatures and corresponding change in expression of *FGFR1* and *FGF23* were further validated in PMT tissues from a test cohort of another three TIO patients. In summary, we reported the first study of the mutational landscape and genetic signatures of cfDNA in TIO/PMTs.

## 1 INTRODUCTION

Tumour‐induced osteomalacia (TIO), also known as oncogenic osteomalacia, is a rare paraneoplastic syndrome characterized by hypophosphataemia due to renal phosphate wasting, inappropriately normal or low 1,25‐dihydroxy vitamin D, and either elevated or inappropriately normal plasma FGF23 level.^1^ Tumour‐induced osteomalacia patients are always presented with certain non‐specific symptoms, such as bone pain, fractures, muscle weakness and, occasionally, a reduction in height. The tumours inducing this syndrome are mostly benign phosphaturic mesenchymal tumours (PMTs) of variable sizes, which can locate anywhere in the body.^2^, ^3^ Previous studies revealed that more than half of PMTs are in the extremities, but almost 40% are not in the limbs,^4^ making them difficult to locate. Behaviourally, even though this tumour commonly infiltrates the surrounding connective tissue, it rarely metastasizes. Nowadays, various combinations of functional and anatomical imaging are used for clinical diagnosis, including selective venous sampling for FGF23 measurements,^5^ computed tomography (CT) scans, magnetic resonance imaging (MRI),^6^ octreotide scintigraphy,^7^ fluorodeoxyglucose (FDG)^8^ and Gallium‐68 DOTATATE positron emission tomography (PET)/CT.^9^

However, the mechanisms underlying the tumorigenesis of PMTs and its secretion of those phosphatonins remain obscure. A relevant finding in understanding its tumorigenesis was the identification of fibronectin 1 (*FN1*) and fibroblast growth factor receptor‐1 (*FGFR1*) translocations which led to an *FN1‐FGFR1* fusion protein in 60% of studied PMTs by RNA sequencing or FGFR‐specific fluorescence in situ hybridization (FISH).^10^ Additional studies have confirmed this finding in a larger group of PMTs.^11^ In addition to the description of the *FN1‐FGFR1* translocation, it has been recently reported that 6% of PMTs had an *FN1*‐fibroblast growth factor 1 (*FGF1*) translocation.^11^ However, these findings still suggest the existence of the tumorigenic drivers behind the fusion‐negative cases, and the mutational landscape and genetic signatures of PMTs are yet to be elucidated.

Here, we employed circulating cell‐free DNA (cfDNA) analysis as a non‐invasive liquid biopsy to study the tumorigenesis of TIO. The cfDNAs mainly come from the fragmented genomic DNA (gDNA) of apoptotic, secretory or necrotic cells. Specifically, the cfDNA released by tumour cells was termed as circulating tumour DNA (ctDNA),^12^ which carries tumour‐specific genetic mutations, including single‐nucleotide variations (SNVs), indels, genome rearrangements and copy number variations (CNVs).^12^ With this method, diagnosis and genomic alterations of several cancers have been reported, such as metastatic breast cancer,^13^ lung cancer^14^ and exocrine pancreatic cancer.^15^ In particular, there is a high degree of concordance between the mutation profiles of ctDNA and tumours metastasis.^16^ Breakthroughs have increased the sensitivity of ctDNA detection by next‐generation sequencing (NGS), contributing to early detections and diagnoses for many kinds of tumours.^16^, ^17^, ^18^ Besides, analysis of non‐invasive cfDNA has advantages over traditional biopsies in revealing the heterogeneity compared with tissue biopsies, because of its once‐for‐all capture and detection of mutations present in multiple tumour deposits.^19^ Here, we recruited four TIO patients for cfDNA mutation profiling via an NGS approach targeting 422 cancer‐relevant genes. The TIO‐specific genetic signatures were explored by comparing our sample to cfDNAs of patients with bone metastasis (BM), as the positive control, and that of the healthy (negative) controls (HC). As a proof‐of‐concept study, we offered more information on the cfDNA landscape of TIO and shed a light on the genetic signatures for non‐invasive detection and monitor of TIOs for the first time.

## 2 MATERIALS AND METHODS

### 2.1 Patient enrolment and sample preparation

**Figure 1:**
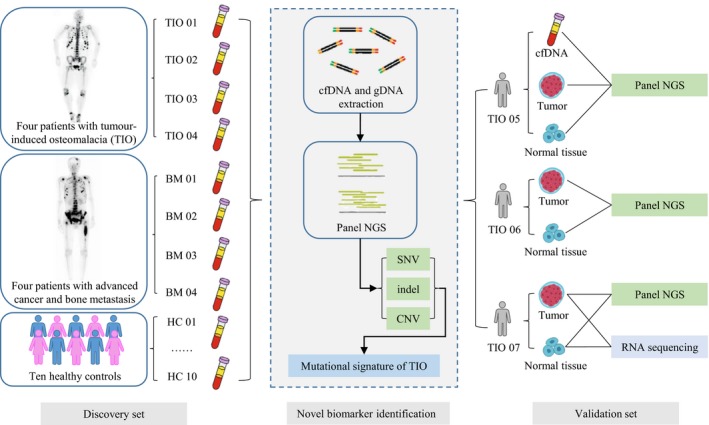
The workflow of the searching biomarker in cfDNA of TIO patients. Blood samples were obtained from four patients with tumour‐induced osteomalacia, four patients with tumour and bone metastasis and ten healthy controls. cfDNA was extracted from blood samples and underwent panel NGS to compare SNV, indel and CNV among three groups so that biomarkers could be found. NGS, next‐generation sequencing; TIO, tumour‐induced osteomalacia; BM, bone metastasis; HC, healthy control; SNV, single‐nucleotide variation; CNV, copy number variation; LoF, loss‐of‐function

Initially, a case‐control design was adopted to identify the genetic signatures of the cfDNAs in TIO and PMT. Then, an additional test cohort of TIO patients was enrolled to validate the previous findings (Figure 1). All participants were enrolled through the Deciphering Disorders Involving Scoliosis & Comorbidities (DISCO) study (http://www.discostudy.org) and the Genetic investigation of Inherited and Familial Tumor Syndrome (GIFTS) study from the Peking Union Medical College Hospital (PUMCH). Patients were eligible for enrolment if they had had evident histologic diagnoses of TIO or BM. The pathology was confirmed by the haematoxylin and eosin staining. The clinicopathological characteristics of all eight patients were retrospectively reviewed. In the validation set, we recruited three more TIO patients and collected their relevant data as well. Written informed consent was obtained from each participant. The study was reviewed and approved by the Ethics Committee of PUMCH.

Ten millilitres of peripheral blood was collected from each patient before surgeries and placed into EDTA‐coated tubes (BD Biosciences), and samples were also obtained from individuals in the control group. Plasma was extracted within the 2 hours following blood collection, and plasma samples were shipped to the central testing laboratory within 48 hours. Normal tissue adjacent to the tumour was collected at a distance of at least 2 cm away from the tumour margin.^20^ Plasma FGF23 was measured using *FGF23* ELISA Kit (Kainos).

### 2.2 Cell‐free DNA purification and quantification

The cfDNA from plasma was extracted using the QIAmp Circulating Nucleic Acid Kit (Qiagen). Genomic DNA from tumour, normal tissue and leucocytes in peripheral blood was extracted with the QIAamp DNA Mini Kit (QIAGEN). Purified genomic DNA was qualified by NanoDrop 2000 for A260/280 and A260/A230 ratios (Thermo Fisher Scientific). All DNA samples were quantified by Qubit 3.0 using the dsDNA HS Assay Kit (Life Technologies). The size distribution of cfDNA was analysed using Bioanalyzer 2100 with the High Sensitivity DNA Kit (Agilent Technologies).

### 2.3 Library preparation

Sequencing libraries were prepared using the KAPA Hyper Prep Kit (KAPA Biosystems) with an optimized manufacturer's protocol. In brief, 1 μg of genomic DNA, which was sheared into 350‐bp fragments using Covaris M220 instrument (Covaris), or 2‐50 ng of cfDNA, underwent end‐repairing, A‐tailing and ligation with indexed sequencing adapters sequentially, followed by size selection for genomic DNA libraries or purification for cfDNA libraries using Agencourt AMPure XP beads (Beckman Coulter). Finally, libraries were amplified by PCR and purified using Agencourt AMPure XP beads.

### 2.4 Target gene panel sequencing and data processing

Different libraries with unique indices were pooled together in desirable ratios for up to 2 μg of total library input. Human cot‐1 DNA (Life Technologies) and xGen Universal blocking oligos (Integrated DNA Technologies) were added as blocking reagents. Customized biotin‐labelled DNA probes (Geneseeq Technology Inc) targeting 422 cancer‐relevant genes were used for hybridization enrichment (listed in Table S1). The capture reaction was performed with Dynabeads M‐270 (Life Technologies) and xGen Lockdown hybridization and wash kit (Integrated DNA Technologies) according to the manufacturers' protocols. Captured libraries were on‐beads PCR amplified with Illumina p5 (5′ AAT GAT ACG GCG ACC ACC GA 3′) and p7 primers (5′ CAA GCA GAA GAC GGC ATA CGA GAT 3′) in KAPA HiFi HotStart ReadyMix (KAPA Biosystems), followed by purification using Agencourt AMPure XP beads. Libraries were quantified by qPCR using the KAPA Library Quantification Kit (KAPA Biosystems). Library fragment size was determined by Bioanalyzer 2100 (Agilent Technologies). Enriched libraries were sequenced using the Illumina HiSeq 4000 platform to reach the mean coverage depth of 3000× for each sample.

Paired‐end sequencing data were aligned to the reference human genome (build hg19) with the Burrows‐Wheeler Aligner (bwa‐mem).^21^ Alignment results (BAM files) were further processed for de‐duplication, base quality recalibration and indel realignment using the Picard suite^22^ (http://broadinstitute.github.io/picard) and the Genome Analysis Toolkit (GATK).^23^ VarScan2^24^ was employed for the detection of SNVs and insertion/deletion mutations (indels) with the following parameters: minimum read depth = 50, minimum base quality = 20, minimum variant supporting reads = 5, variant supporting reads mapped to both strands and strand bias no greater than 10%. Mutations were removed if they were present in >1% population frequency in the 1000 Genomes Project, ExAC or 6500ESP databases. The resulted mutation lists were further filtered through an internally collected list of 10 normal samples in the HC groups (~3000× depth) on the same sequencing platform. Specifically, if a variant was detected, that is ≥4 mutant reads and in ≥2 of the normal samples, it was considered a likely artefact of common germline mutations and was removed. SNV and indel annotation was performed by ANNOVAR^25^ using the hg19 reference genome and 2014 versions of standard databases and functional prediction programmes. Only protein‐coding mutations were kept, while loss‐of‐function (LoF) mutation was defined as damaging mutations of frameshift indel, start‐loss, stop‐gained and stop‐loss SNVs. The rare mutation was defined as the mutation presented less than 0.1% in common populations. The germline variants were curated and interpreted according to the guideline of ACMG (American College of Medical Genetics and Genomics),^26^ by using variant prediction tool InterVar.^27^

### 2.5 RNA extraction and library construction

Total RNA from frozen samples was extracted using the RNeasy Mini Kit (QIAGEN). Ribosomal RNA was depleted using RNase H followed by library preparation using KAPA Stranded RNA‐seq Kit with RiboErase (HMR) (KAPA Biosystems). Library concentration was determined by KAPA Library Quantification Kit (KAPA Biosystems), and library quality was accessed by Agilent High Sensitivity DNA Kit on Bioanalyzer 2100 (Agilent Technologies), which was then sequenced on Illumina HiSeq NGS platforms (Illumina).

### 2.6 Gene expression analysis

Base calling was performed on bcl2fastq v2.16.0.10 (Illumina) to generate sequence reads in FASTQ format (Illumina 1.8+ encoding). Quality control (QC) was performed with Trimmomatic (version 0.33).^28^ STAR (version 2.5.3a)^29^ is used for transcriptome mapping followed by isoform and gene‐level quantification performed by RSEM (version 1.3.0).^30^ Differential expression analysis was conducted by R packages DESeq2 (version 1.16.1)^31^ and edgeR (version 3.18.1).^32^ Differentially expressed genes were selected by fold change >2 and *P* < .05. Corresponding volcano plots and heatmaps were generated by in‐house R scripts.

### 2.7 Fusion detection

Three tools were applied for fusion detection of RNA‐seq data. FusionCatcher^33^ (version 0.99.4e) was used with parameters that use Bowtie^34^ aligner to perform both transcriptome and genome mapping and then use BLAT^35^ aligner to further map unmapped reads and count fusion supporting evidence. The other two tools namely FACTERA^36^ and Socrates (https://github.com/jibsch/Socrates) were both executed using default parameters. Especially, the Socrates takes the modified BAM file, which converted hard‐clip in original BAM into soft‐clip to improve the fusion detection performance. The combined fusion results from all tools were manually reviewed on IGV^37^ for confirmation.

### 2.8 Gene ontology analysis

To gain biologically functional insights of the gene clusters identified in TIO‐only, BM‐only and TIO&BM‐both group, we conducted gene set enrichment analysis using R (version 3.4.3; https://www.r-project.org/). For annotation, the biological process terms of the Gene Ontology (GO) project^38^, ^39^ were involved as the biological knowledge, and the clusterProfiler package^40^ was used for over‐representation test. All biological process terms with *P* value adjusted <.01 were retained for further analysis. For reducing the functional redundancy of enriched terms, we calculated the similarities among GO terms and remove those highly similar terms by choosing the most representative term with the GOSemSim package.^41^ For data visualization, only the top ten most enriched terms were presented.

### 2.9 Statistical analysis

The somatic variants were identified by comparing the variants of cfDNA and tumours to the normal tissue or leucocytes in peripheral blood. With the starting cut‐off point of 0.5% mutant allele frequency (MAF), comparisons among TIO, BM and HC were able to attain by statistical analysis. Association analysis was performed for allelic and genotypic association utilizing the SPSS software v15.0 (SPSS). The difference of MAFs and mutation types among different groups was compared using the chi‐square test. The characteristics were presented as means ± standard deviations. Odds ratio (OR) with 95% confidence interval (CI) was used to assess the difference of mutation types in the cfDNA in three groups. The two‐sided *P < *.05 was considered as statistically significant.

## 3 RESULTS

### 3.1 Clinical characteristics of the patients with tumour‐induced osteomalacia and bone metastasis

To investigate the TIO‐specific genetic signatures, we concurrently utilized both positive and negative control groups. Four bone metastasis patients were recruited as positive controls considering that their clinical performance was similar to TIO and cfDNA was reported to be good liquid biopsy for detecting advanced cancer.^12^, ^13^ Another 10 unrelated healthy individuals whose gender and age were matched with the patients in the TIO and BM groups were considered as negative controls to exclude non‐specific mutations and systematic errors (Figure 1). As the discovery cohort, two female and two male TIO patients with a mean age of 40 ± 10 years and one female and three male BM patients with a mean age of 49 ± 13 years were enrolled (Table 1). The clinical symptoms included bone pain starting from different parts of the body such as back and lower limbs. However, CT/MRI and intraoperative exploration showed the tumour locations varied a lot. The phosphorus levels in the blood of all the TIO patients in the discovery and the validation cohort were low before tumours removed. The blood phosphate of most patients increased to the normal level in the post‐operate follow‐up. Only one patient TIO01 who had her tumour removed still had hypophosphataemia postoperatively, even after a 1‐year follow‐up (Table 1). Also, the TmP/GFR (ratio of the renal tubular maximum reabsorption rate of phosphate to glomerular filtration rate) also decreased, indicating the reduction of renal reabsorption of phosphorus. We also obtained the elevated *FGF23* levels of these seven patients before operation, ranging from 62.83 to 374.67 pg/mL comparing to the normal level of 10‐50 pg/mL.^42^ The pathological results indicated that tumours of TIO patients were all PMTs (Figure S1) and the locations of all the lesions were also been confirmed, consistently with our previous study.^3^ In this study, all the PMTs were around or inside the bone, but in different positions, including the medial right shoulder joint, left lateral femoral condyle, L5 left pedicle and left distal ulna and radius. In addition, the corresponding primary tumours of the BM group are shown in Table 1.

**Table 1:** The clinical information of the patients in this study

Patient	Gender	Age	Pathology of primary tumour	Pain start	Location of tumour	Pre‐surgery					P‐discharge (mmol/L)	P‐One‐week follow‐up (mmol/L)	FGF23 level (pg/mL)
						TmP/GFR (mmol/L)	Ca^2+^ (mmol/L)	PTH (pg/mL)	1,25(OH)2D3 (pg/mL)	P (mmol/L)			
The discovery set													
TIO01	F	27	Phosphaturic mesenchymal tumour	Right foot	Medial right shoulder joint	0.2	2.15	98.5	6.99	0.28	0.42	0.44	71.5
TIO02	M	51	Phosphaturic mesenchymal tumour	Right foot	Left lateral femoral condyle	NA	2.38	35.8	40.04	0.52	0.99	1.63	62.8
TIO03	F	38	Phosphaturic mesenchymal tumour	Right leg	L5 left pedicle	0.45	2.10	76.7	5.98	0.34	0.91	1.09	374.7
TIO04	M	42	Phosphaturic mesenchymal tumour	Back	Left distal ulna and radius	0.6	2.35	45.0	29.49	0.58	0.85	1.02	79.9
BM01	M	51	Renal clear cell carcinoma	Left leg	L2 left pedicle	NA	2.97	NA	NA	NA	NA	NA	NA
BM02	F	32	Breast invasive ductal carcinoma	Right femur	Right femoral cavity	NA	2.24.	NA	NA	NA	NA	NA	NA
BM03	M	50	Melanoma	Left leg	L1‐L4 spinous process	NA	2.09	NA	NA	NA	NA	NA	NA
BM04	M	63	Colon cancer	Back	L3‐L5 spinous process	NA	2.05	NA	NA	NA	NA	NA	NA
The validation set													
TIO05	M	53	Phosphaturic mesenchymal tumour	Left rib	Right ankle joint	0.5	2.27	114.1	13.33	0.50	0.81	0.94	365.2
TIO06	M	28	Phosphaturic mesenchymal tumour	Right rib	Right proximal tibia	0.48	2.33	45.6	26.80	0.56	0.91	1.08	84.3
TIO07	M	64	Phosphaturic mesenchymal tumour	Lower limb and neck	Left medial popliteal fossa	0.48	2.16	116	NA	0.64	0.75	1.01	261.1

Abbreviations: 1,25(OH)2D3, the active form of vitamin D, normal range of 19.6‐54.3 pg/mL; BM, bone metastasis; Ca^2+^, blood calcium, normal range of 2.13‐2.70 mmol/L; FGF23, fibroblast growth factor 23, normal range of 10‐50 pg/mL; L1, the 1st lumbar vertebra; NA, not available; P, blood phosphate, normal range of 0.81‐1.45 mmol/L; PTH, parathyroid hormone, normal range of 12.0‐65.0 pg/mL; S1, the 1st sacral vertebra; TIO, tumour‐induced osteomalacia; TmP/GFR, ratio of tubular maximum reabsorption rate of phosphate to glomerular filtration rate, normal range of 0.88‐1.44 mmol/L.

### 3.2 Quality of the sequencing data

In the DNA sample set of eight patients and ten healthy controls, we used an NGS approach: high coverage panel genes for detection of both sequence and structural alterations (Figure 1). The average coverage of each base in the targeted regions was 2015 ± 252‐fold (range, 1724‐ to 2448‐fold) in cfDNA. Thus, we were able to cover over 99.95% of target bases. With this high coverage analysis, we identified 4994 ± 654 (range, 4156‐5914) genomic variants in these samples and a few CNVs in one TIO sample and two BM samples (Table S2).

### 3.3 The mutational landscape of cfDNA in the tumour‐induced osteomalacia and bone metastasis groups

**Figure 2:**
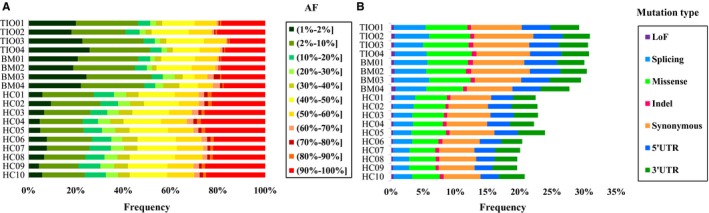
Bar diagram based on allele frequencies (AF) and mutation types. In A and B diagrams, no significant difference was observed between TIO and BM groups, while the HC group was much different from both the TIO and BM groups. A, Allele frequencies ranging from 1% to 100% were divided into eleven isolated intervals. All the mutation variants were divided into eleven groups based on allele frequencies. B, All the mutation variants were divided into seven groups based on mutation types: LoF, splicing, missense, indel, synonymous, 5′UTR and 3′UTR. TIO, tumour‐induced osteomalacia; BM, bone metastasis; HC, healthy control; LoF, loss‐of‐function; UTR, untranslated region; AF, allele frequency

To comprehensively analyse genetic differences among the three groups, we compared the distributions of the allele frequencies (AF) in three groups (Figure 2A). Interestingly, there was no obvious difference between the TIO and BM groups (*P* = .946). Nevertheless, the differences between the case groups (TIO and BM) and the control group (HC) were both extremely significant (*P* < 10^−100^).

In addition to frequency distribution analysis, the mutation types were also analysed. Similarly, TIO and BM were closely alike (*P* = .495) and HC varied widely (*P = *3.05 × 10^−58^ and *P* = 7.55 × 10^−50^, respectively) (Figure 2B). More specifically, there were significantly more LoF mutations (*P* = .048, OR 1.351, 95% CI 1.002‐1.822), splicing mutations (*P* = 5.97 × 10^−22^, OR 1.629, 95% CI 1.474‐1.801), missense mutations (*P* = 1.24 × 10^−15^, OR 1.410, 95% CI 1.295‐1.534), synonymous mutations (*P* = 8.60 × 10^−14^, OR 1.331, 95% CI 1.234‐1.435) and mutations in 5′UTR (*P* = 5.94 × 10^−8^, OR 1.320, 95% CI 1.194‐1.460) in the TIO group than the HC group except 3′UTR (*P* = .098) and indel (*P* = .304). On the other hand, BM and HC varied in LoF mutations (*P* = .017, OR 1.427, 95% CI 1.064‐1.914), splicing mutations (*P* = 2.45 × 10^−26^, OR 1.700, 95% CI 1.540‐1.877), missense mutations (*P* = 5.30 × 10^−9^, OR 1.295, 95% CI 1.187‐1.413), synonymous mutations (*P* = 3.58 × 10^−7^, OR 1.223, 95% CI 1.131‐1.321), mutations in 5′UTR (*P* = 1.10 × 10^−8^, OR 1.339, 95% CI 1.211‐1.480) and mutations in 3′UTR (*P* = .039, OR 1.112, 95% CI 1.005‐1.229) except indel (*P* = .169).

### 3.4 Exploring the genetic signatures of tumour‐induced osteomalacia in cfDNA

**Figure 3:**
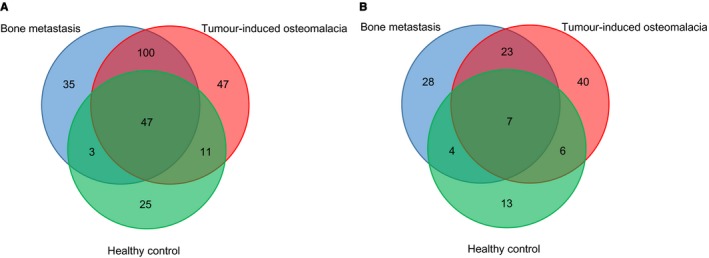
Venn diagrams representing mutation genes with LoF and rare missense in three groups. A, Numbers of unique and shared genes with rare missense mutations in TIO, BM and HC groups. B, Numbers of unique and shared genes with LoF mutations in the three groups. More mutations were detected in rare missense A than LoF B. Common gene variants of the three groups take a big part in both rare missense A and LoF B. TIO, tumour‐induced osteomalacia; BM, bone metastasis; LoF, loss‐of‐function

To identify the potentially deleterious mutations, we focused on the functional somatic variants, including rare missense and LoF mutations. The somatic variants were identified by comparing the variants of cfDNA to the gDNA from the peripheral blood. With the strict exclusion criteria, we next identified and compared statistically significant mutations among TIO, BM and HC groups (Figure 3, Tables S3 and S4). Generally, more mutation genes of rare missense were detected than that of LoF. Meanwhile, only seven shared genes of LoF between TIO and BM were identified. There were 47 unique rare missense mutations for TIO and 35 for BM (Figure 3A), and 40 LoF mutations for TIO and 28 for BM, respectively (Figure 3B).

**Figure 4:**
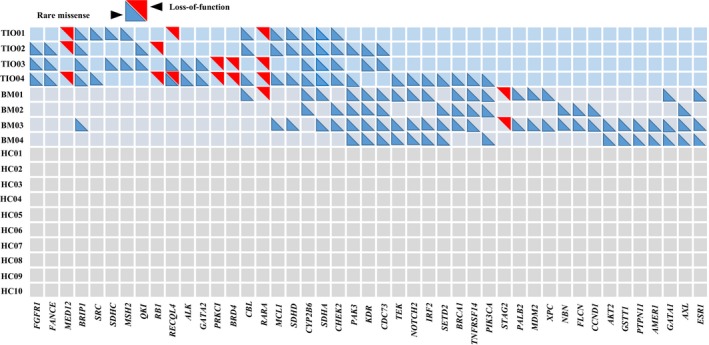
Distributions of 45 mutation genes of rare missense and loss‐of‐function in eight patients. Minimally overlapping of mutation genes was shown between TIO and BM patients. The number of mutation genes playing a major role in TIO patients is 21, and the number of mutation genes in BM patients is 24. TIO, tumour‐induced osteomalacia; BM, bone metastasis

We then analysed the detailed distributions of the above pathogenic mutations. We selected candidate genes in which mutation distribution was different between TIO and BM by over 50% (no <2 in 4 cases). With such screening criteria, we further narrowed down the mutations to 45 mutation genes, including 39 rare missense mutations and seven LoF mutations (Figure 4, Table S5). These genes showed rarely overlapping between TIO and BM patients. Rare mutations within *FGFR1* (c.2018G>A in TIO02 and TIO03 and c.668A>T in TIO04, Table S5)*, FANCE* (c.1321A>T in TIO02, c.80G>A in TIO03 and c.128G>A in TIO04, Table S5) and *MED12* (c.1080delT in TIO01, c.103G>T and c.4901delA in TIO02, and c.2827delC and c.1080delT in TIO04, Table S5) were found in three of four TIO patients (75%), while *BRIP1* mutations were found in all the TIO patients (100%) and one of four BM patients (c.1934A>T and c.1916A>T in TIO01, c.1934A>T in TIO02, c.1918A>T in TIO03, c.1916A>T in TIO04, and c.1934A>T and c.1916A>T in BM03, Table S5). Likewise, rare mutations within *GATA1* (c.32C>T in BM01, c.622G>A in BM03 and c.1175G>A in BM04, Table S5)*, AXL* (c.157A>T in BM02 and BM03, c.1752T>A and c.71A>C in BM04, Table S5) and *ESR1* (c.1236G>T in BM01, c.1236G>T and c.442G>A in BM03, and c.1236G>T in BM04, Table S5) were found in three of four BM patients but none in TIO patients.

### 3.5 Gene ontology enrichment analysis revealing biological processes responding to tumour‐induced osteomalacia and bone metastasis

**Figure 5:**
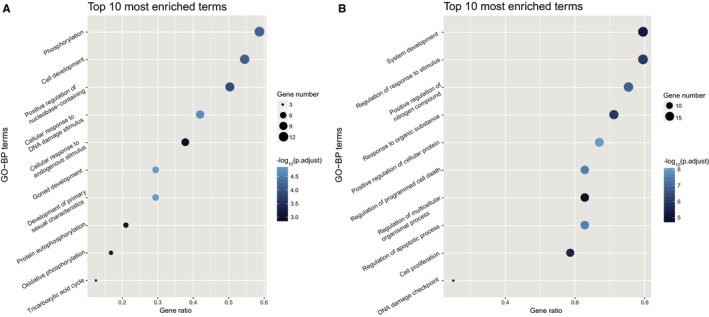
Gene ontology (GO) biological processes of the intersection sub‐network in TIO and BM. Bubble diagrams of the gene ontology biological processes of the intersection sub‐network in TIO and BM based on specific mutation genes were mapped using stats package in R environment. A, The most outstanding biological process focused on phosphorylation. B, Most of the biological processes focused on the regulation of cancer survive and migration. The bubble size indicated the number of genes enriched in the corresponding process, and the colours of the bubbles demonstrated the *P* value. TIO, tumour‐induced osteomalacia; BM, bone metastasis

To verify the specific functions the genes related to the TIO and BM patients may involve in the disease process, we conducted an enrichment analysis for GO related to biological processes to gain insights into the molecular mechanisms. As shown in Figure 5A, among the top ten processes enriched by 21 genes of the TIO group, the most significant biological process was phosphorylation, so did another two processes which involved protein auto‐phosphorylation and oxidative phosphorylation. These biological processes present unique functions in the TIO process. As for another GO enrichment of 24 genes from the BM group (Figure 5B), most of the biological processes related to the regulation of response to the stimulus, programmed cell death and apoptotic process with high enrichment scores. These biological processes were essential for metastatic cancer to survive in migration.^43^ Besides, common processes showed that cell communication and cell proliferation were important for both disease development (Figure S2).

### 3.6 Validating the genetic signatures of cfDNA in phosphaturic mesenchymal tumours

To validate the reliability of the genetic signatures which were previously found in the cfDNA of patients with TIO, a prospective cohort of three more TIO patients (TIO05, TIO06 and TIO07) was enrolled. The TmP/GFR rate and the blood phosphorus level of these patients were also low before the surgery. The corresponding primary tumours are shown in Table 1.

For comparing the consistency of the variants in cfDNA and primary tumours, the cfDNA from plasma, the tumour DNA from the PMT and the genome DNA from normal tissue adjacent to the tumour in the patient TIO05 were extracted and sequenced through a panel‐based NGS approach. The somatic variants were identified by comparing the variants of cfDNA and PMT to the normal tissue. Among 269 somatic variants in cfDNA and 214 somatic variants in PMT of TIO05, 79 variants (29.37% for cfDNA and 36.92% for the PMT) were shared between the cfDNA and the primary tumour (Table S7). There were 86 variant genes shared in cfDNA and tumour in TIO05, accounting for 86/147 (58.50%) and 86/116 (74.14%) variant genes in cfDNA and PMT, respectively (Table S7). Two in‐frame deletion variants within *MED12* (c.6165_6167delGCA and c.6165_6167delGCA) were shared in cfDNA and PMT. However, a frameshift variant within *MED12* (c.5285delA) was only found in cfDNA with an AF of 0.43%, and two *MED12* frameshift variants which were previously found in the TIO patients in the discovery cohort (c.1080delT in TIO01 and TIO04, and c.4901delA in TIO02) were identified only in the PMT tissue of TIO05 with the AFs less than 1%. Additionally, a missense variant within *FGFR1* (c.239G>A) with an AF of 0.25% was only found in cfDNA but not in PMT.

To identify the origin of the deleterious mutations of *FGFR1* and *MED12* in cfDNA, the DNA from the PMT tissues and normal tissues of the three TIO patients in the validation cohort was sequenced using the NGS panel targeting 422 cancer‐relevant genes (Table S1). As a result, intriguingly, the *FGFR1* missense variant (c.239G>A) which was found in cfDNA of TIO05 was also found in somatic variants in the PMT tissues in TIO06 and TIO07 with relatively AFs of 0.16% and 29%, respectively. However, the LoF variant of *MED12* was only found in the PMT tissue of TIO05. The germline variants were also curated according to the ACMG guideline,^26^ while no deleterious mutation was identified for these three patients (Table S8).

To illustrate the correspondence changes in the transcriptome of PMT to the genetic signatures, the gene expression analysis of a pair of the PMT tissue and normal tissue of patient TIO07 was conducted by total RNA sequencing. Comparing to the normal tissue, the expression of *FGF23*, *FGF1* and *FGFR1* was all significantly up‐regulated with the Log2FoldChange of 5.97, 3.22 and 1.78, respectively, and the *P* value of 5.80 × 10^−38^, 2.18 × 10^−21^ and 2.19 × 10^−6^, respectively (Figure S3). In addition, several gene fusions including the *FN1‐FGFR1* fusion were identified (Table S9). Similarly, to better understand the underlying biological processes of gene expression alterations, the functional enrichment analysis was performed based on the top 200 most significant differentially expressed genes. The functional enrichment analysis suggested that these differentially expressed genes mainly participated in bone‐associated biological processes, such as ossification, skeletal system development, which was consistent with the top ten processes enriched from mutated genes of the cfDNA of the TIO patients in the discovery cohort (Figure S4).

## 4 DISCUSSION

Our study aimed to elucidate the mutational landscape and the genetic signatures of TIO in cfDNA. While the PMTs responsible for TIO are typically benign, metastasis can also occur.^1^ By and large, TIO, to some extent, resembles malignant tumours.^44^ Though there are a few fusion genes findings, the mechanisms underlying the tumorigenesis of PMTs and its phosphatonins secretion have not been fully elucidated. NGS of plasma cfDNA might be a promising strategy. Other studies in ctDNA analysis, including identification of drug resistance, have been conducted.^45^ As far as we know, most of the studies about cfDNA focused on the malignant cancers, which makes our report the first study concerning cfDNA and its mutational landscape in benign tumours and rare diseases. The current findings provided proof‐of‐concept evidence for the possibility of non‐invasive detection of PMT and TIO.

According to previous studies, the variant allele frequency (VAF) and the function of mutations are essential features for the variants in cfDNA.^46^, ^47^ To reveal the mutational landscape of cfDNA, we first compared the comprehensive differences in the distributions of both allele frequencies and the types of mutations among TIO/PMT patients, patients with bone metastasis (positive control) and healthy participants (negative controls). Intriguingly, we found that the malignant and benign tumours shared many similar genetic signatures in genes, AFs and mutation types. The cfDNAs contain DNA fragments from both normal cells and tumour cells in the stages of apoptosis, secretion, or necrosis, and those DNA fragments carried tumour‐specific genomic alterations. Previous studies have also shown that an isolated benign tumour cell could not be distinguished from an isolated malignant tumour cell.^48^ Genetically, cfDNA also failed to discriminate between malignant and benign breast lesions.^49^ To avoid the false positivity, large cohorts were recommended.^49^ A recent study reported that endometriotic lesions, commonly considered to be benign inflammatory lesions, exhibited cancer‐like features such as local invasion and resistance to apoptosis, which harboured some specific somatic cancer driver mutations.^50^ Therefore, our findings were not only consistent with those previous studies but also extended the spectrum of similarities between malignant and benign tumours to shared mutations and mutational landscape. For example, we identified 79.46% (147/185) genes with rare missense mutations in the BM group and 71.71% (147/205) in the TIO group were shared (Figure 3A, Table S4). For LoF mutations, there were 48.39% (30/62) genes with LoF mutations in the BM group and 39.47% (30/76) of the TIO group shared the same locus of mutation (Figure 3B, Table S4). Taken together, 82.93% (170/205, Table S4) mutation genes involved in BM might also be involved in TIO, probably participating in neoplastic progression, affecting cell cycle, tumour growth and DNA repair.

We also reported the genetic signatures of TIO. The initial candidate CNVs, due to their uneven distributions and unclear functions, could not be very important to TIO. However, their related genes together with other mutations found in this study showed highly relevant biological functions in TIO progression by GO analysis. The first was a rare *FGFR1* missense found in 3 of 4 TIO patients which was predicted pathogenic by SIFT and PolyPhen‐2 (Table S5). The protein encoded by *FGFR1* is a member of the fibroblast growth factor receptor (FGFR) family, in which the amino acid sequence is highly conserved among its members and throughout evolution. It is the best‐established receptor required for *FGF23* signalling. *FGF23* acts primarily on renal proximal tubular cells, leading to decreased renal phosphate reabsorption 42 and calcium absorption.^1^ Binding by its co‐receptor Klotho, *FGF23* activates the mitogen‐activated protein kinase (MAPK) pathway, which can regulate cell proliferation, survival and *FGF23* secretion.^51^ In our gene expression analysis of the PMT of TIO07, the expression of *FGF23*, *FGF1* and *FGFR1* was all significantly up‐regulated, which indicated that the *FGFR1* mutation might play a role in PMT genesis, though further functional studies are needed. Therefore, these biological processes facilitating the phosphorylation of FGFR1 might contribute to the secretion of *FGF23* and thus play a part in the TIO process. The recurrent *FGFR1* mutation has been reported in various neoplastic disorders 52and some rare diseases like osteoglophonic dysplasia, which is an extremely rare bone dysplasia exhibiting an autosomal dominant mode of inheritance.^53^, ^54^, ^55^ In our study, only one patient still had hypophosphataemia after surgical removal of the tumour, and her condition did not resolve based on her 1‐year follow‐up. Interestingly, this patient was the only TIO patient without *FGFR1* mutation in cfDNA***.***

In the validation set, the fusion of *FN1* and *FGFR1* was identified in the gene expression analysis of the PMT from TIO07. A relevant finding in the study of PMT pathogenesis identified *FN1* and *FGFR1* translocations as a leading cause of *FN1‐FGFR1* fusion protein in 60% of samples by RNA sequencing or fluorescence in situ hybridization (FISH).^11^ The *FN1‐FGFR1* fusion gene would presumably be highly expressed since FN1 is an ubiquitously expressed extracellular protein and is driven by a strong promoter.^10^ In this way, *FGF23* secretion would be up‐regulated to down‐regulate the levels of phosphate and other biomolecules associated with PMTs.

Meanwhile, *MED12* was also found to be possibly involved in the process of TIO/PMT. MED12 protein is a subunit of the mediator which can function in transcriptional activation or repression. The defect of *MED12* has been reported to be associated with other types of tumours, such as prostate cancer and breast fibroepithelial tumours.^56^ Besides, *MED12* mutations can also interfere with bone development in some congenital diseases, like Opitz‐Kaveggia syndrome characterized by relative macrocephaly.^57^ A recent study reported that in vivo deletion of *Med12* caused rapid bone marrow aplasia that resulted in acute death, proving that *MED12* was also an essential regulator of hematopoietic stem cell homeostasis.^58^ In addition, many studies have reported that *MED12* mutations were abundant in benign tumours like uterine leiomyomas^59^ and breast fibroadenoma^60^ that were both exclusively located in the stroma. However, owing to our small sample size and *MED12*'s low allele frequency, we could not confirm its role in TIO.

The other two mutated genes, *FANCE* and *BRIP1*, though without evident clinical reports, still showed interesting results. *FANCE* mutations were found in three of four TIO patients and none of the BM patients. *BRIP1* mutations were found in all of the TIO patients (100%) and one of the four BM patients. *FANCE* is included in the Fanconi anaemia complementation group essential for DNA damage repair. Defects in this gene can cause Fanconi anaemia, a disorder with clinical features of cytopaenia, bone developmental defects and tumour predisposition.^61^ *BRIP1* is a DEAH helicase that interacts with the BRCT repeats of *BRCA1* and has an important role in *BRCA1*‐dependent DNA repair. It can function independently of *BRCA1* in the Fanconi anaemia pathway for DNA crosslink repair and is defective in some Fanconi anaemic patients.^62^

For the BM group, we also found some relevant mutated genes which might have functions yet awaits to be discovered. Rare mutations in *GATA1, AXL* and *ESR1* were found in three of the four BM patients. *GATA1* is a prototypical lineage‐restricted transcription factor that is central to the normal differentiation, proliferation, and apoptosis of erythroid and megakaryocytic cells. Mutations in *GATA1* can contribute to the genesis of transient myeloproliferative disorder and acute megakaryoblastic leukaemia.^63^ The protein encoded by *AXL* is a member of the Tyro3‐Axl‐Mer receptor tyrosine kinase subfamily. The increased expression of *AXL* in cancer acts as a mechanism of acquired drug resistance, frequently accompanied by epithelial‐to‐mesenchymal transition.^64^ *ESR1* encodes oestrogen receptor 1, which can initiate or enhance gene transcription in response to oestrogen stimulation. Oestrogen has a multifunctional role in affecting the growth, differentiation and functions of different tissues, which is reported to be activated in hormone‐resistant bone‐metastatic breast cancer.^65^ Oestrogen also has an important role in regulating bone growth and bone remodelling in adults. Mutations on oestrogen are believed to be associated with severe osteoporosis.^66^

The major limitation of our study is its relatively small sample size, as TIO is a rare disease. Validations in larger cohorts are still needed. Additionally, due to the fact that PMTs responsible for the osteomalacia were small in size, there was not enough tumour mass leftover for DNA or RNA sequencing after routine pathological diagnoses. Also, the target gene strategy prevented us from finding all the mutations, CNVs and gene fusions within the genome. Finally, the exact functions of *FGFR1* and *MED12* mutations need to be confirmed by further functional studies in cell lines and animal models, which would confer much greater knowledge base for this rare disease.

In conclusion, we reported the first study of the mutational landscape and genetic signatures of cfDNA in TIO and PMT. Mutations such as *FGFR1* might have important clinical implications. As a pioneer study, this cfDNA analysis represents a unique opportunity in the studies of rare tumours genetics and gives some reference for the future studies.

## CONFLICT OF INTEREST

The authors have no conflict of interests to declare. The raw data that support the findings of this study are openly available in the supplementary materials.

## AUTHORS' CONTRIBUTIONS

NW, Yang S, JZ, WX, GQ, Yong L, Jiaqi L and ZW designed and conducted this research. ZZ, Xi Z, WC, NG, YY, Ying L, WL, Jia L, NZ, Xu Y and YX recruited the participants in this study. HZ, YM, XW, Xian Z, Xin‐Zhuang Y, MZ, HB, YW, YZ and JS analysed and interpreted the data. HW, YN and Yalun L performed the experiments. NW, ZZ, Xi Z, HZ, Yong L, Jiaqi L and ZW wrote the draft manuscript. All authors contributed to the writing of the manuscript and approved the final manuscript for submission.
