# Supplementary figures and images for: Mutational landscape and genetic signatures of cell‐free DNA in tumour‐induced osteomalacia
Source: J Cell Mol Med. 2020 Apr 11;24(9):4931–43. doi: 10.1111/jcmm.14991 (PMC7205804; doi:10.1111/jcmm.14991)

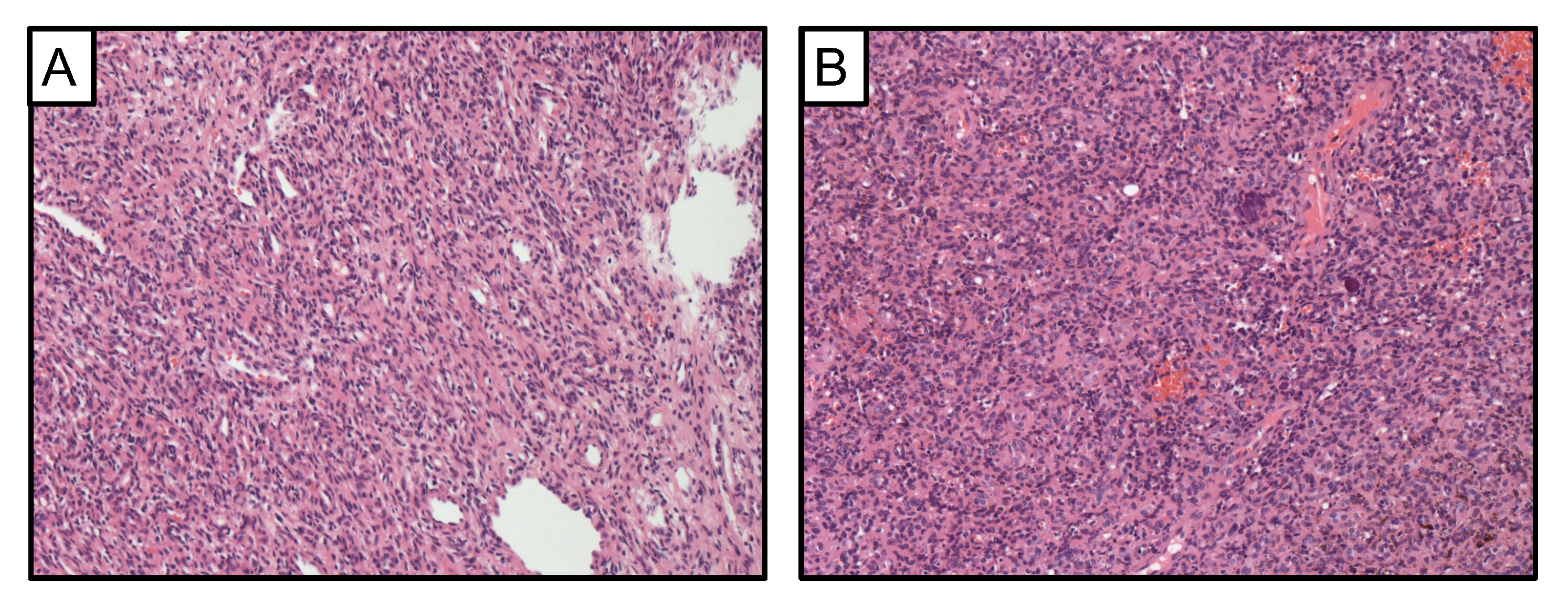

Supplement: Supplementary file 1 [file JCMM-24-4931-s001.tiff]

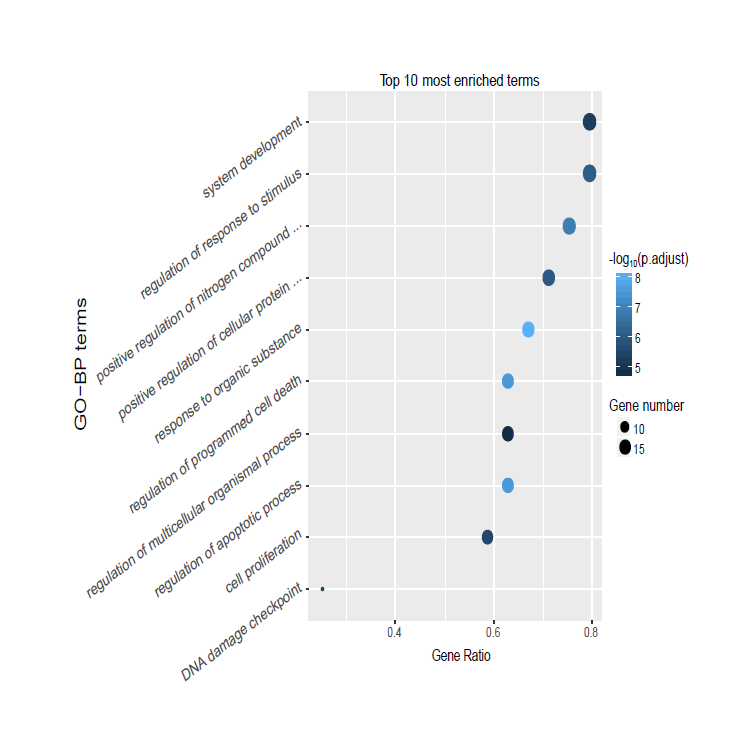

Supplement: Supplementary file 2 [file JCMM-24-4931-s002.tiff]

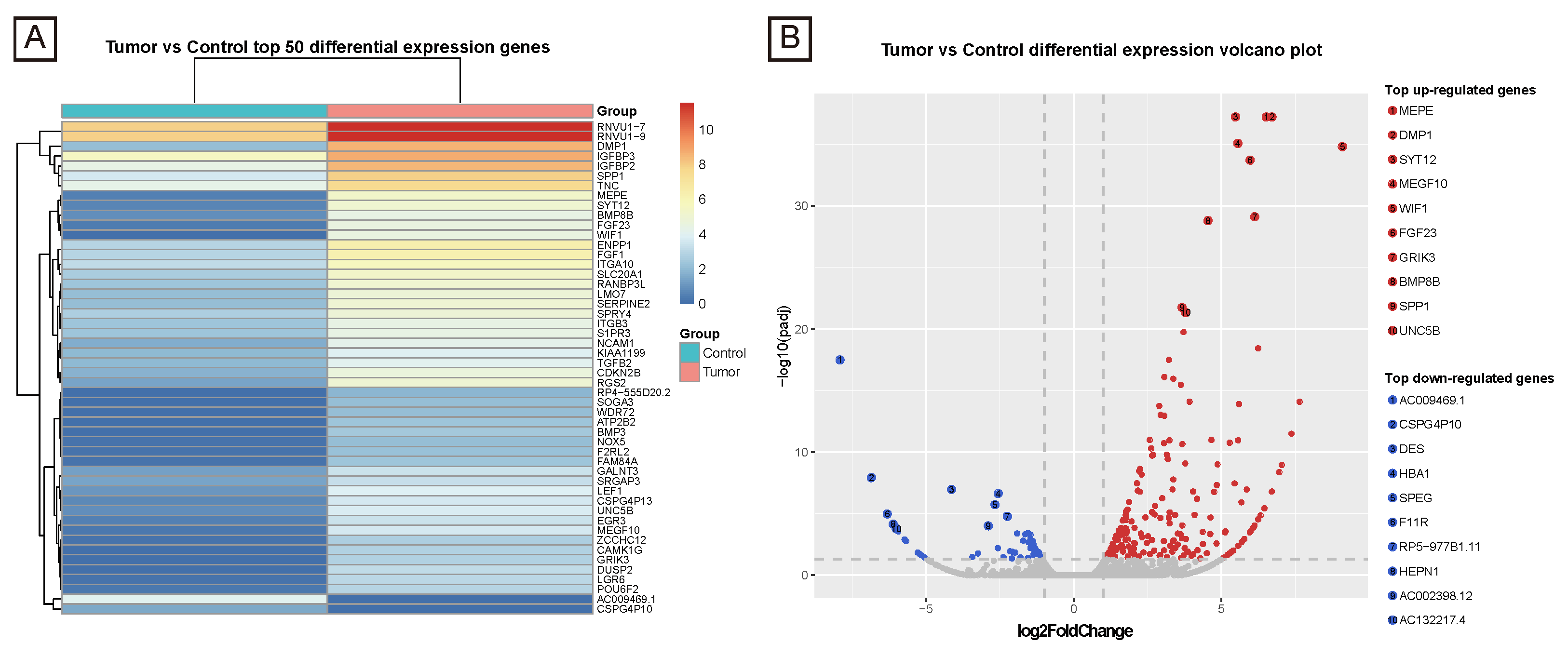

Supplement: Supplementary file 3 [file JCMM-24-4931-s003.tif]

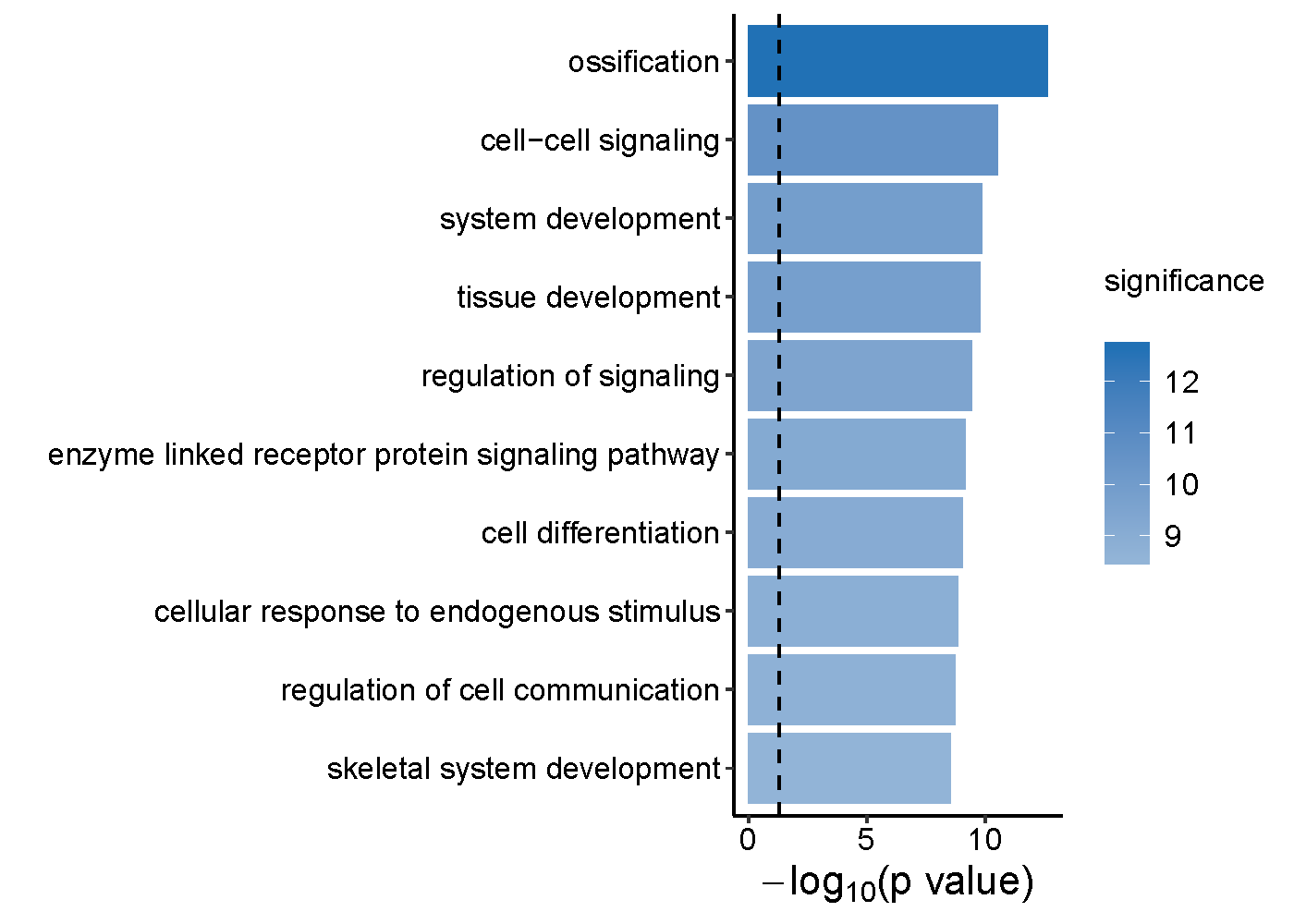

Supplement: Supplementary file 4 [file JCMM-24-4931-s004.tif]
