# Supplementary material for: Mutational landscape and genetic signatures of cell‐free DNA in tumour‐induced osteomalacia
Source: J Cell Mol Med. 2020 Apr 11;24(9):4931–43. doi: 10.1111/jcmm.14991 (PMC7205804; doi:10.1111/jcmm.14991)
Supplement: Supplementary file 6 [file JCMM-24-4931-s006.docx]

# Table S2. CNV Detection in the ctDNA of Patients.

| **Patient** | **TIO01** | **TIO02** | **TIO03** | **TIO04** | **BM01** | **BM02** | **BM03** | **BM04** |
| --- | --- | --- | --- | --- | --- | --- | --- | --- |
| CNV/Amplification* | N.A. | N.A. | GSTT1 / 2.2 | N.A. | N.A. | N.A. | MET / 8.7  GRM3 / 2.1  POT1 / 2.1 | MITF / 1.7 |

Abbreviation: CNV, copy number variations; Amplification, gene amplification times.
