# Supplementary material for: Mutational landscape and genetic signatures of cell‐free DNA in tumour‐induced osteomalacia
Source: J Cell Mol Med. 2020 Apr 11;24(9):4931–43. doi: 10.1111/jcmm.14991 (PMC7205804; doi:10.1111/jcmm.14991)
Supplement: Supplementary file 7 [file JCMM-24-4931-s007.docx]

# Table S3. The Mutational Landscape of Variants in cfDNA in the Three Groups

| **Characteristics** | **Tumor-induced osteomalacia** | **Bone metastasis** | **Healthy controls** | **TIO *vs* BM*** | | **TIO *vs* HC*** | | **BM *vs* HC*** | |
| --- | --- | --- | --- | --- | --- | --- | --- | --- | --- |
|  |  |  |  | ***p*** | **OR (95%CI)** | ***p*** | **OR (95%CI)** | ***p*** | **OR (95%CI)** |
| LoF mutations | 58 | 61 | 168 | 0.766 | 1.056 (0.737-1.514) | 0.048 | 1.351 (1.002,1.822） | 0.017 | 1.427 (1.064,1.914） |
| Splicing mutation | 558 | 579 | 1359 | 0.482 | 0.958 (0.851-1.079) | 5.97×10^-22^ | 1.629 (1.474-1.801) | 2.45×10^-26^ | 1.700 (1.540-1.877) |
| Missense mutation | 766 | 704 | 2157 | 0.114 | 1.088 (0.980-1.209) | 1.24×10^-15^ | 1.410 (1.295-1.534) | 5.30×10^-9^ | 1.295 (1.187-1.413) |
| Indel | 76 | 79 | 260 | 0.790 | 1.044 (0.761-1.431) | 0.304 | 1.144 (0.885-1.478) | 0.169 | 1.194 (0.927-1.536) |
| Synonymous mutation | 964 | 887 | 2879 | 0.078 | 1.088 (0.990-1.196) | 8.60×10^-14^ | 1.331 (1.234-1.435) | 3.58×10^-7^ | 1.223 (1.131-1.321) |
| 5’UTR | 522 | 527 | 1559 | 0.824 | 0.986 (0.872-1.115) | 5.94×10^-8^ | 1.320 (1.194-1.460) | 1.10×10^-8^ | 1.339 (1.211-1.480) |
| 3’UTR | 498 | 506 | 1793 | 0.749 | 0.980 (0.864-1.111) | 0.098 | 1.089 (0.984-1.205) | 0.039 | 1.112 (1.005-1.229) |
| Rare missense mutation | 205 | 185 | 86 | 0.327 | 0.905 (0.741-1.105) | 9.22×10^-99^ | 9.320 (7.240-11.996) | 3.02×10^-86^ | 8.551 (6.617-11.051) |

*The difference of mutation types among different groups were compared using the Chi-square test. The two-sided *p<*0.05 was considered as statistically significant.

Abbreviation: TIO, tumor-induced osteomalacia; BM, bone metastasis; HC, healthy controls; LoF, loss-of-function; OR, odds ratio; CI, confidence interval.
